# When cure becomes an illness—abuse of addictive prescription medicines

**DOI:** 10.3389/fphar.2015.00193

**Published:** 2015-09-15

**Authors:** Mihajlo (Michael) Jakovljevic, Marija Lazarevic, Milena Jurisevic, Mirjana R. Jovanovic

**Affiliations:** ^1^Faculty of Medical Sciences, University of KragujevacKragujevac, Serbia; ^2^Department of Pharmacy, Faculty of Medical Sciences, University of KragujevacKragujevac, Serbia; ^3^Department of Psychiatry, Faculty of Medical Sciences, University of KragujevacKragujevac, Serbia; ^4^Psychiatry Clinic, University Clinical Center KragujevacKragujevac, Serbia

**Keywords:** addiction, drugs of abuse, pharmaceutical market, expenditure, trend, costs

## Substance addiction as societal phenomenon—the case of Serbia

Psychoactive substance addiction presents a challenging public health issue worldwide particularly targeting vulnerable adolescent population (Babor et al., 2007). The illegal market turnover of the controlled substances is very difficult to trace and intervene timely among the young in the early stage of illness (Inciardi et al., 2007). Contrary to this one, legally regulated pharmaceuticals pose another dangerous threat that is more accessible to the authorities. In order to combat substance dependence the national strategies should specially emphasize prescription medicines with proven potential of abuse and addiction (Compton and Volkow, 2006).

Serbia as the largest Western Balkans market and a typical Eastern European transitional health system might serve as an appropriate example of medicines abuse in the EU borderlands (Jakovljevic, 2013). The results of local research in 2008, showed that 15.1% of first grade high school students, at least once had used, some psychoactive substance (excluding nicotine and alcohol), while 7.6% of them used sedatives, which was the most common [European School Survey Project on Alcohol and Other Drugs (ESSPAD), 2008]. The national survey on life styles of citizens in Serbia in 2014 about substance use and gambling addiction reported that in the past 12 months 22.4% of the respondents used sedatives, anxiolytics or hypnotics drugs (13.9% of males and 30.9% of females) and in the last 30 days 14.6% of respondents (8% of males and 21.2% of females) used these drugs with higher preponderance among older population. In the last 12 months 5.1% of the respondents used opioids for pain treatment (4.1% of males and 6.1% of females) and in the last 30 days 2.2% of respondents (1.6% of males and 2.8% of females) used these drugs. A large number of respondents said that they had purchased these drugs in the pharmacies prescribed by their physicians –85.3% of the population 18–64 years of age used hypnotics and sedatives, and 70.3% of the population used opioid drugs (Kilibarda et al., 2014).

Various studies have shown that drug addiction disorders are related to personality disorders, anxiety disorders and a considerably higher suicide rate (Regier et al., 1990). Further consequences of drug addiction are: education (Yamada et al., 1993) and employment underachievement, reduced work productivity, poor health, higher rates of human immunodeficiency virus (HIV) and hepatitis B and C infections (Jakovljevic et al., 2013a), social dysfunction, higher rate of violence and incarceration among that population, poverty, homelessness, a lower probability of recovery, poor treatment outcomes, and poor quality of life. The treatment system for substance use disorders is accompanied by high public and private expenses (Jovanovic and Jakovljevic, 2011).

## Pharmaceutical market in a late-stage transitional health reform setting

The national health care system of Serbia is financed by mandatory tax contributions by employers and employees alike to the National Health Insurance Fund. The health care system of Serbia similar to Eastern European countries, have undergone profound transitional reforms with different degrees of success in different areas of medical service provision and outcomes (Jakovljevic et al., 2011). Over the last 20 years, pharmaceutical and total health care expenditures were growing more rapidly than gross domestic product (GDP) in many world regions (Getzen, 1990). The Serbian pharmaceutical market was characterized by a large growth in public expenditure on pharmaceuticals (from €339,279,304 in 2004 to €794,560,045 in 2013). The Sudden expansion in the market led to the increase of Serbia's public debt and accumulated debt of wholesalers toward brand industry in the entire region (Jakovljevic et al., 2015a). These developments eventually led to the crisis of financial sustainability and serious market shortages of even some fundamental medicines (Jakovljevic, 2014). Primary health care facilities were intended to act as “gate keepers” of the health care system. Their success in this role to contain “supplier induced demand” in clinical medicine was frequently examined in public opinion (Vuković et al., 2015). Preventive public health measures and early screening procedures remain an ongoing weakness of the most regional health systems and Serbia is no exception. In the long run, the evolution of prosperity diseases and accelerated population aging are most likely to persist as the core challenges in the broader Balkans region (Jakovljevic and Laaser, 2015).

## Signs of addictive prescription medicines abuse

Published trials from late 1990s provide clear-cut evidence of upward trend in the consumption of psychiatric drugs in Serbia and Montenegro (Miljkovic and Djukic, 2000). The Utilization of all drugs acting on the nervous system (inclusive of psychiatric drugs) grew from 28.9 DDD/1000 inhabitants/day in 1996 to 69.1 DDD/1000 inhabitants/day in 2004. Among the causes of this growth, the most frequently cited are increased incidence of psychiatric disorders, post-war syndromes that followed the Yugoslav civil wars and higher accessibility of prescription medicines at the time (Divac et al., 2004).

Contemporary policy, since the early 2000s, is that majority of approved and marketed potentially addictive medicines are assigned to the positive list and completely reimbursed within the basic health insurance premium. Extensive annual reports on prescription and dispensing of drugs in terms of defined daily doses per 1000 inhabitants per day (DDD/1000 inhabitants/day) are released at regular times by the National Medicines and Medicinal Devices Agency of Serbia (ALIMS; Agencija za lekove i medicinska sredstva Srbije, 2013). In Table 1 we can remark DDDs and value base turnover of six selected ATC (Anatomical Therapeutic Chemical Classification System)^1^ code groups of such medicines: anesthetics, opioids, anxiolytics, hypnotics and sedatives, psychostimulants, agents used for ADHD (Attention deficit hyperactivity disorder), nootropics and drugs used in addictive disorders. The results are presented using WHO recommended ATC/DDD methodology (Sjöqvist and Birkett, 2003). The consumption of psychostimulants, agents used for ADHD and nootropics grew tremendously (from 0.35 DDD/1000 inhabitants/day in 2004 to 6.61 DDD/1000 inhabitants/day in 2013). The consumption of hypnotics and sedatives has almost doubled (from 2.47 DDD/1000 inhabitants/day in 2004 to 4.02 DDD/10000 inhabitants/day in 2013) primarily due to benzodiazepine derivatives. A promising sign is the rise of prescription of drugs used to treat addictive disorders (from 0.31 DDD/1000 inhabitants/day in 2004 to 1.50 DDD/1000 inhabitants/day in 2013). Among these ATC code groups, the most frequent consumption was recorded in the case of agents that are indicated in opioid dependence.

**Table 1:** **National level dispensing (Defined Daily Doses per 1000 population/day) and consumption (€) of major potentially addictive medicines and those used to treat substance addiction in 2004 and 2013 in Serbia**.

**ATC code group**	**Reimbursed value of dispensed medicines in 2004**	**Reimbursed value of dispensed medicines in 2013**	**DDD per 1000 inhabitants/hospital day in 2004**	**DDD per 1000 inhabitants/hospital day in 2013**
**POTENTIALLY ADDICTIVE MEDICINES**				
N01A Anesthetics, General	€1,283,128	€4,072,544	N/A	N/A
N01AF Barbiturates, plain	€21,465	€101,842^2009^	N/A	N/A
N02A Opioids	€1,472,459	€2,499,864	0.84	0.52
N02AA Natural opium alkaloids	€197,341	€611,410	0.04	0.08
N02AB Phenylpiperidine derivatives	€272,205	€1,341,574	0.03	0.11
N02AD Benzomorphan derivatives	€818	€506^2006^	0.00	N/A
N02AX Other opioids (mostly tramadol based preparations)	€1,002,095	€546,880	0.77	0.33
N03AA Barbiturates and derivatives	€427,676	€263,570	2.9	2.03
N03AE Benzodiazepine derivatives	€291,568	€1,288,405	0.63	2.48
N05AH Diazepines, oxazepines, thiazepines, and oxepines	€1,529,558	€4,925,668	0.37	1.53
N05B Anxiolytics	€9,450,094	€15,091,052	106.95	79.81
N05BA Benzodiazepine derivatives	€9,448,412	€15,091,052	106.95	79.81
N05C Hypnotics and sedatives	€1,197,390	€2,232,897	2.47	4.02
N05CD Benzodiazepine derivatives	€1,197,390	€1,459,522	2.47	1.98
N05CF Benzodiazepine related drugs	€0	€773,375	0.00	2.03
N06B Psychostimulants, agents used for ADHD and nootropics	€287,766	€3,138,433	0.35	6.61
R01AA Sympathomimetics, plain (mostly ephedrine based preparations)	€1,676,145	€4,328,551	132.14	14.83
R05DA Opium alkaloids and derivatives	€74,118	€28,649	0.05	0.03
R06AB Substituted alkylamines (mostly pheniramine derivatives)	€492,461	€2,764,304	0.56	0.08
**MEDICINES USED TO TREAT SUBSTANCE ADDICTION**				
N07B Drugs used in addictive disorders	€236,734	€1,900,777	0.31	1.50
N07BA Drugs used in nicotine dependence	€138,139	€407,718	0.03	0.10
N07BB Drugs used in alcohol dependence	€35,123	€206,726	0.19	0.53
N07BC Drugs used in opioid dependence	€63,472	€1,286,332	0.09	0.88

Pharmaceutical spending related to all of the aforementioned groups of medicines has strongly increased during the same time span. The most significant budget impact was attributed to the anxiolytics consumption in 2013 (€15,091,052) in comparison to €9,450,094 back in 2004. This finding is wholly justified by previous findings on heavily increased anxiety levels among resident addicts in the former Yugoslav region compared to traditional EU-15 market economies (Jakovljevic et al., 2013b). Psychostimulants related costs were multiplied 10-fold (€287,766 in 2004 to €3,138,433 in 2013). Over the same time horizon following aforementioned growth pattern in DDD terms, the costs of pharmaceuticals used to treat substance dependence increased heavily as well (from €236,734 in 2004 to €1,900,777 in 2013).

## Conclusive remarks

Although the situation appears to implicate an extensive public debate in this region, we should be aware of the fact that a large portion of reported consumption is clinically justified with basic mental illness. It would be highly difficult and demanding rigorous methodological approach to exactly identify the proportion of overutilization of these drugs that is attributed to abuse and addiction. Nevertheless, vast net quantity gains in consumed addictive medications in definite ATC groups by far surpass extended burden of associated psychiatric morbidity in recent years (Ristic-Ignjatovic et al., 2013). Therefore, this insight gives grounds for serious concerns about widespread patient abuse of addictive medicines in this country and beyond.

The abuse of potentially addictive medicines correlates with clinicians' decision making principles (Bendtsen et al., 1999) and locally prevailing psychiatrist practice (Jakovljevic et al., 2015b). Citizens' possibility to purchase these medicines without medical prescription has been proven as another major driver of abuse potential (Lessenger and Feinberg, 2008). It is reported that up to 4.4% adults consuming sedatives and hypnotics on a daily base. This amounts to an overall population of 182,000–234,000 regular users of sedatives and hypnotics among population aged from 18 to 64. This phenomena might have a difficult and concerning extent among the small Balkan nations (Divac et al., 2009). Similar life styles reported in public health surveys across EU might indicate that the abuse of potentially addictive medicines could have the far reaching consequences in the European continent (United Nations Office on Drugs and Crime, 2010). There are several possible tactics to cope with this challenge for overprescribing pattern and irrational consumption of these medicines could be partially controlled by paying more attention to potentially susceptible patients during regular check-ups (Khan et al., 2015). Strengthening of the regulatory framework in marketing approval and reimbursement could impose more strict legal control to the over-the-counter sales of this medicines. Software based good clinical practice guidelines have already confirmed strong benefits in terms of patient outcomes and cost savings in a local clinical setting (Jakovljevic et al., 2014). Health education program development targeted for addictive patients' needs (Wolde et al., 2008) and those suffering from other mental illnesses at the risk of developing substance dependence, could improve patient support and counseling (Hashemi and Hashemi, 2015). The experience of the successful implementation of alternative and complementary medicine methods attained in many countries, might serve as an additional example of exit strategies probably to bring relief to this vulnerable patient population.

## Funding

The underlying studies providing evidence for this contribution where funded out of The Ministry of Education Science and Technological Development of the Republic of Serbia Grant OI 175014. Publication of results was not contingent to Ministry's censorship or approval.

### Conflict of interest statement

The authors declare that the research was conducted in the absence of any commercial or financial relationships that could be construed as a potential conflict of interest.

## Footnotes

^1^Anatomical Therapeutic Chemical Classification System. Available online at: http://www.whocc.no/atc_ddd_index/.
